# Tubulin Polymerization Promoting Protein, Ringmaker, and MAP1B Homolog Futsch Coordinate Microtubule Organization and Synaptic Growth

**DOI:** 10.3389/fncel.2019.00192

**Published:** 2019-05-15

**Authors:** Qian Shi, Yong Qi Lin, Afaf Saliba, Jing Xie, G. Gregory Neely, Swati Banerjee

**Affiliations:** ^1^Department of Cellular and Integrative Physiology, Long School of Medicine, University of Texas Health, San Antonio, TX, United States; ^2^The Dr. John and Anne Chong Lab for Functional Genomics, Charles Perkins Centre and School of Life and Environmental Sciences, The University of Sydney, Sydney, NSW, Australia; ^3^Xiangya School of Medicine, Central South University, Changsha, China

**Keywords:** Ringmaker, TPPP, Futsch, microtubule, acetylation, synapse

## Abstract

*Drosophila* Ringmaker (Ringer) is homologous to the human Tubulin Polymerization Promoting Proteins (TPPPs) that are implicated in the stabilization and bundling of microtubules (MTs) that are particularly important for neurons and are also implicated in synaptic organization and plasticity. No *in vivo* functional data exist that have addressed the role of TPPP in synapse organization in any system. Here, we present the phenotypic and functional characterization of *ringer* mutants during *Drosophila* larval neuromuscular junction (NMJ) synaptic development. *ringer* mutants show reduced synaptic growth and transmission and display phenotypic similarities and genetic interactions with the *Drosophila* homolog of vertebrate Microtubule Associated Protein (MAP)1B, *futsch.* Immunohistochemical and biochemical analyses show that individual and combined loss of Ringer and Futsch cause a significant reduction in MT loops at the NMJs and reduced acetylated-tubulin levels. Presynaptic over-expression of Ringer and Futsch causes elevated levels of acetylated-tubulin and significant increase in NMJ MT loops. These results indicate that Ringer and Futsch regulate synaptic MT organization in addition to synaptic growth. Together our findings may inform studies on the close mammalian homolog, TPPP, and provide insights into the role of MTs and associated proteins in synapse growth and organization.

## Introduction

The establishment of proper neuronal connectivity in the nervous system is central to our cognition and behavior. Neuronal connections in the form of synapses are stabilized through trans-synaptic adhesion complexes (Mosca et al., 2012; Banerjee et al., 2017; Südhof, 2017) that are anchored in the underlying cytoskeleton (Koch et al., 2008). A major component of the neuronal cytoskeleton is the microtubule (MT) network that provides structural support for the growing axons (Kapitein and Hoogenraad, 2015). The polymerization of MTs and their bundling and stabilization are critical for formation, extension and guidance of axons (Lewis et al., 2013). While the regulation of MT organization and dynamics has been well-studied during axon and dendrite formation and maintenance, much less is known about MT dynamics in synapses.

A number of proteins and associated factors maintain the dynamic nature of MTs (Conde and Caceres, 2009; Akhmanova and Steinmetz, 2010). Classical microtubule-associated proteins (MAPs) perform a wide range of functions to regulate MT dynamics for properly organizing and remodeling the neuronal cytoskeleton (Ramkumar et al., 2018). All MAP1 proteins play a role in MT stabilization and their transfection in heterologous cells results in the formation of MT bundles (Noiges et al., 2002). MAP1 family, particularly MAP1B has distinct roles in MT-based processes such as, neuronal migration, growth cone turning and actin-based processes such as dendritic spine maturation (Meixner et al., 2000; Tortosa et al., 2011; Bodaleo et al., 2016). Although several MAPs have been identified, the mechanisms underlying coordinated regulation of axonal cytoskeleton with synaptic growth remain to be fully elucidated. The only *Drosophila* homolog of vertebrate MAP1B, Futsch, is necessary for dendritic, axonal and synaptic MT organization and growth (Hummel et al., 2000; Roos et al., 2000). Futsch controls neuromuscular junction (NMJ) bouton growth through regulation of synaptic MTs (Roos et al., 2000). Recent studies on *Drosophila futsch* have uncovered its role in stabilizing active zones (AZs) by reinforcing their link with the underlying MT cytoskeleton and in regulating neurotransmitter release at the NMJ (Lepicard et al., 2014) as well as activity dependent AZ remodeling of photoreceptor synapses (Sugie et al., 2015).

Apart from the classical MAPs, there is also a unique family of highly conserved but not well-understood tubulin-binding proteins called the Tubulin Polymerization Promoting Proteins (TPPPs) that are implicated in the modulation and coordination of dynamics and stability of the MT network (Hlavanda et al., 2002; Tirián et al., 2003; Oláh et al., 2013) and display extensive MT bundling and polymerization abilities (Hlavanda et al., 2002; Lehotzky et al., 2010; Tokési et al., 2010; Zotter et al., 2011; Mino et al., 2016; Szabó et al., 2017). In addition to MT bundling activity, TPPPs have been implicated in regulating the levels of acetylation of MT network (Tokési et al., 2010; Szabó et al., 2017). Acetylation of MTs is evolutionarily conserved and represents one of the diverse post-translational modifications conferred to MTs. Although biochemical and *in vitro* studies linking TPPP to MT network are known, no mammalian *in vivo* knockout studies have been reported so far to address its role in nervous system development and function. Recently, from a large scale forward genetic screen, we discovered the *Drosophila* homolog of human TPPP named Ringmaker (Ringer; Mino et al., 2016). Ringer is a major regulator of axonal MT organization and functions in axon extension (Mino et al., 2016). Similar to Ringer, TPPP in zebrafish has been implicated in axon outgrowth (Aoki et al., 2014; Orosz, 2015). While a recent report described TPPP localization in nerve terminals of mice and human retina (Tripon et al., 2018), the role of Ringer or any TPPP family of proteins in other species in regulating MT organization during synaptic development and function remains to be elucidated.

Here, we report the first *in vivo* phenotypic and functional characterization of Ringer in presynaptic MT organization and synaptic growth at the NMJs. *ringer* mutants have synaptic undergrowth, ultrastructural anomalies and reduced synaptic transmission. Interestingly, *ringer* mutants display phenotypic similarities with *futsch* mutants and also show genetic interactions. Furthermore, Ringer and Futsch exist in a biochemical complex and regulate acetylation and stability of MTs. Our studies underscore the importance of MT dynamics in proper synaptic organization and growth and identify a highly conserved cytoskeletal protein family, TPPP/Ringer that regulate these processes with the MAP1B/Futsch. Together our studies will provide insights into similar interactions that might be used by vertebrates in regulating synaptic growth and architecture.

## Results

### Ringer Localizes at the Presynaptic NMJ Terminals and Is Required for Bouton Growth

**FIGURE 1:**
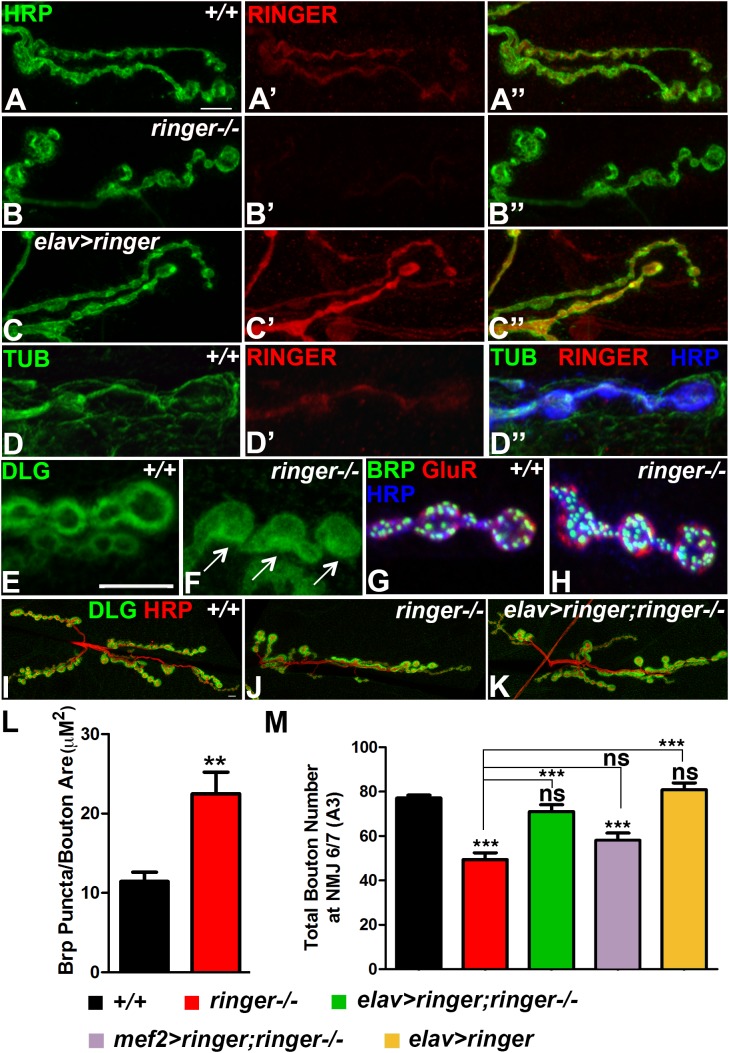
Ringer localizes to the presynaptic terminals and is required for synaptic growth. **(A–C″)** Confocal images showing Ringer localization (red) in wild type **(A′,A″)**, *ringer–/–* **(B′,B″)** and *elav-Gal4;UAS-ringer* **(C′,C″)** with respect to the neuronal Hrp (green, **A,A″,B,B″,C,C″**) at the presynaptic terminals. **(D–D″)** Ringer expression **(D′,D″)** with respect to Tub (green, **D,D″**) and Hrp (blue, **D″**) at the wild type NMJ. Dlg localization in wild type **(E)** and *ringer–/–* **(F)** NMJ. Apposition of AZs labeled with Brp (green, **G,H**), GluRIIA (red, **G,H**) and Hrp (blue, **G,H**) in wild type **(G)** and *ringer–/–* **(H)**. Synaptic arbor labeled with Dlg (green, **I–K**) and HRP (red, **I–K**) in wild type **(I)**, *ringer–/–* **(J)** and presynaptic rescue in *elav-Gal4;UAS-ringer;ringer–/–* **(K)**. **(L,M)** Quantification of Brp puncta/bouton area (μM^2^) in wild type and *ringer–/–* **(L)** and total Type 1 boutons in specified genotypes **(L)**. *n* = 15 animals per genotype, Error bars represent mean ± SEM [^∗^*p* ≤ 0.05, ^∗∗^*p* ≤ 0.01, ^∗∗∗^*p* < 0.001, not significant (ns)] (ANOVA). Scale bars: **(A–D″)** = 5 μM; **(E–H)** = 5 μM; **(I–K)** = 10 μM.

Our recent studies on Ringer revealed that it is expressed in embryonic, larval, and adult nervous system (Mino et al., 2016). We wanted to further investigate what role Ringer plays in the larval nervous system. We examined the subcellular localization of Ringer by co-staining third instar larvae with anti-Ringer (red, Figure 1A′,A″–C′,C″) and the neuronal marker Hrp (green, Figure 1A,A″–C,C″). Since endogenous Ringer was expressed at very low levels, we increased the brightness of the images equally across all genotypes (Figure 1A–D″) post-imaging to evaluate Ringer expression more clearly. Ringer localized to the presynaptic NMJ terminals in the wild type (Figure 1A′,A″) and, as expected, was absent in *ringer* null mutant NMJs (Figure 1B′,B″). Upon presynaptic overexpression in *elav-Gal4;UAS-ringer* (Figure 1C′,C″), Ringer expression was significantly elevated in the presynaptic compartments, thus being more clearly visible at the NMJ boutons. Ringer localized close to the synaptic plasma membrane localization of Hrp (Figure 1A″,C″). Since Ringer is the *Drosophila* homolog of the human TPPP family of proteins, we next examined whether Ringer displayed any colocalization with Tubulin at the NMJ. Ringer showed endogenous expression that overlapped with Tubulin at the presynaptic NMJ terminals (Figure 1D′,D″), while Tubulin had a much broader expression labeling MTs both of the pre- and post-synaptic larval cytoskeleton (green, Figure 1D,D″). Ringer and Tubulin were co-stained with Hrp (blue) that labeled the presynaptic NMJ terminals (Figure 1D″).

We next analyzed the subcellular localization of pre- and post-synaptic proteins in *ringer* mutants compared to their wild type counterparts (Figure 1E–H). We first studied the localization of the *Drosophila* homolog of mammalian PSD-95 protein, Discs large (Dlg). In wild type larval NMJ, Dlg is enriched at the subsynaptic reticulum (SSR) of Type 1b boutons (Budnik et al., 1996) and is excluded from the core of the boutons (Figure 1E). *ringer* mutants showed a more diffuse Dlg distribution throughout the bouton (arrows, Figure 1F). We next studied the distribution of the presynaptic active zone (AZ) protein, Bruchpilot (Brp, Wagh et al., 2006) (green, Figure 1G,H) with respect to the post-synaptic glutamate receptor fields labeled by GluRIIA (Marrus et al., 2004) (red, Figure 1G,H) in wild type (Figure 1G) and *ringer* mutants (Figure 1H) together with Hrp (blue, Figure 1G,H). We did not find any gross abnormalities with the apposition of Brp and GluRIIA puncta in *ringer* mutants (Figure 1H) compared to wild type (Figure 1G) or in the ratio of Brp/GluR IIA puncta between the two genotypes. However, there was a significant increase in the number of Brp-positive puncta as quantified and normalized to the bouton area in *ringer* mutants compared to wild type (Figure 1L). There was also a significant increase in the GluR IIA puncta similar to the Brp puncta in *ringer* mutants compared to the wild type (data not shown).

Next we investigated the consequences of loss of Ringer in NMJ growth (Figure 1I–K,M). We assayed the NMJ arbor by staining larvae of various genotypes (as indicated in Figure 1M) with anti-Dlg (green, Figure 1I–K) and anti-Hrp (red, Figure 1I–K). *ringer* mutants showed significant reduction in the growth of synaptic boutons (Figure 1J,M) compared to wild type (*+/+*, Figure 1I,M). We wanted to check if the synaptic growth phenotype in *ringer* mutants could be rescued by pre- or post-synaptic expression of full length Ringer. We found that synaptic growth in *ringer* mutants was rescued to wild type levels upon presynaptic expression of full length Ringer using the pan-neuronal *elav-Gal4* driver as seen in *elav-Gal4; UAS-ringer; ringer-/-* (Figure 1K,M). Rescue of synaptic growth phenotype in *ringer* mutants, however, could not be achieved by expressing Ringer using the muscle driver as seen in *mef2-Gal4; UAS-ringer; ringer-/-* (Figure 1M) suggesting that Ringer function is presynaptic in the larval NMJ. Presynaptic overexpression of wild type Ringer did not show any significant difference in the bouton count compared to wild type (Figure 1M). Together these studies show that Ringer is pre-synaptically required for proper synaptic organization and growth at the NMJ.

### *Ringer* Mutants Display Defects in Synaptic Ultrastructure and Show Reduced Synaptic Transmission

**FIGURE 2:**
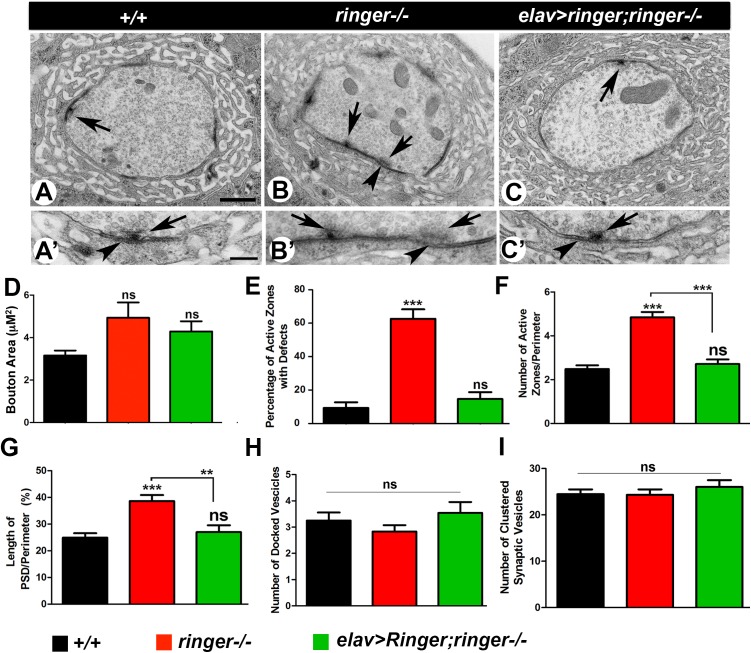
Loss of Ringer show synaptic ultrastructural defects. **(A–C′)** TEM analysis of cross sections through Type 1b boutons at low magnification **(A–C)** and high magnification **(A′–C′)** of wild type **(A,A′)**, *ringer–/–* **(B,B′)** and *elav-Gal4;UAS-ringer; ringer–/–* **(C,C′)**. The AZ T-bars are indicated by arrows and PSD by arrowheads. **(D–H)** Quantification of total bouton area **(D)**, percentage of AZs with defects **(E)**, number of AZs **(F)**, length of PSD **(G)**, number of docked vesicles **(H)**, and number of clustered vesicles **(I)** in represented genotypes. *n* = 50 boutons from five animals of each genotype, Error bars represent mean ± SEM [^∗^*p* ≤ 0.05, ^∗∗^*p* ≤ 0.01, ^∗∗∗^*p* < 0.001, not significant (ns)] (ANOVA). Scale bars: **(A–C)** = 600 nm and **(A′–C′)** = 200 nm.

The reduced size of the AZs and the altered localization of Dlg in *ringer* mutants suggested that loss of Ringer is likely to be associated with structural changes at the NMJ boutons. In order to test for any changes in bouton structure, we next performed transmission electron microscopy (TEM) to examine the ultrastructure of *ringer* mutant boutons (Figure 2B,B′) in comparison with wild type (Figure 2A,A′) and its presynaptic rescue (Figure 2C,C′). For ultrastructural studies, Type 1b boutons from various genotypes were analyzed and subjected to morphometric analyses (Chen et al., 2012; Banerjee et al., 2017). The wild type boutons are characterized by distinct pre- and post-synaptic specializations including AZs composed of electron dense T-bars (arrow, Figure 2A,A′) and the post-synaptic membrane network, the SSR. Our analysis did not show any significant difference in the overall area of the boutons in wild type, *ringer* mutants and *elav-Gal4;UAS-ringer;ringer-/-* (Figure 2D). It is important to note that although many *ringer* mutant boutons had larger bouton areas but there was also a significant variability. *ringer* mutant boutons displayed a range of presynaptic defects including a significantly higher percentage of malformed AZs (arrows, Figure 2B,B′, quantified in E) frequently showing an increase in the width and curvature of the pedestal of T-bars, increased number of AZs (arrows, Figure 2B, quantified in F) and increased length of post-synaptic densities (PSD, arrowheads, Figure 2B,B′, quantified in G) compared to wild type (Figure 2A,A′,E–G, respectively) and *elav-Gal4;UAS-ringer;ringer-/-* (Figure 2C,C′,E–G, respectively). However, there were no significant differences in the distribution or number of synaptic vesicles, such as docked (Figure 2H) and clustered vesicles (Figure 2I), in the presynaptic terminals of *ringer* mutants compared to wild type and *ringer* rescue. These datasets indicate that Ringer is required for proper presynaptic organization of the synaptic boutons.

**FIGURE 3:**
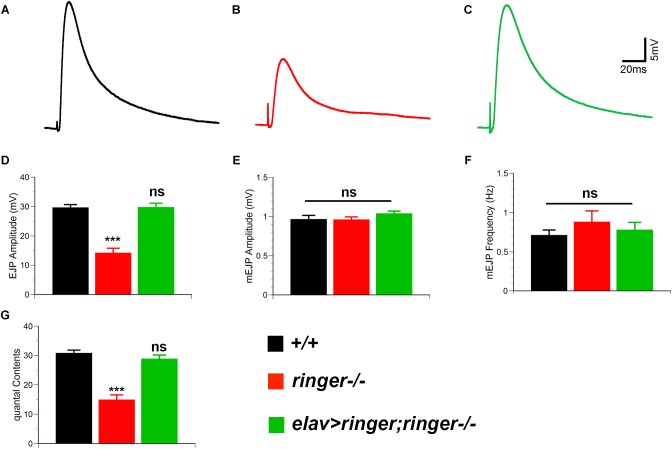
Ringer is required for proper synaptic transmission. Representative electrophysiological traces showing EJPs from wild type **(A)**, *ringer–/–* **(B)**, and *elav-Gal4;UAS-ringer;ringer–/–* **(C)**. Quantification of EJP amplitude **(D)**, mEJP amplitude **(E)**, mini EJP frequency **(F)**, and quantal contents **(G)** in respective genotypes. *n* = 9 (wild type, *+/+*), 9 (*ringer–/–*) and 8 (*elav-Gal4;UAS-ringer;ringer–/–*) recorded animals, Error bars represent mean ± SEM [^∗^*p* ≤ 0.05, ^∗∗^*p* ≤ 0.01, ^∗∗∗^*p* < 0.001, not significant (ns)] (Student’s *t*-test).

Given the ultrastructural deficits in the synapses of *ringer* mutants, we next examined the consequences of loss of Ringer on synaptic transmission at the NMJs. We performed electrophysiological analyses on muscle 6 of third-instar larval body walls and recorded the evoked junctional potentials (EJPs) in 0.5 mM [Ca^2+^] at 0.2 Hz. *ringer* mutants exhibit a reduction in EJP amplitude (Figure 3B,D), compared to wild type (Figure 3A,D) which was rescued in *elav-Gal4; UAS-ringer; ringer-/-* (Figure 3C,D). We observed no significant changes in miniature evoked junctional potentials (mEJP) amplitudes (Figure 3E) and mEJP frequency (Figure 3F) in *ringer* mutants when compared with control wild type and *ringer* rescue genotypes. *ringer* mutants revealed severely decreased quantal contents (Figure 3G) compared with wild type and *ringer* rescue larvae. Together, our data suggest that Ringer is required for proper synaptic organization to allow normal synaptic transmission.

### Ringer and Futsch Co-localize at the Presynaptic NMJ Terminals and Regulate Bouton Growth

**FIGURE 4:**
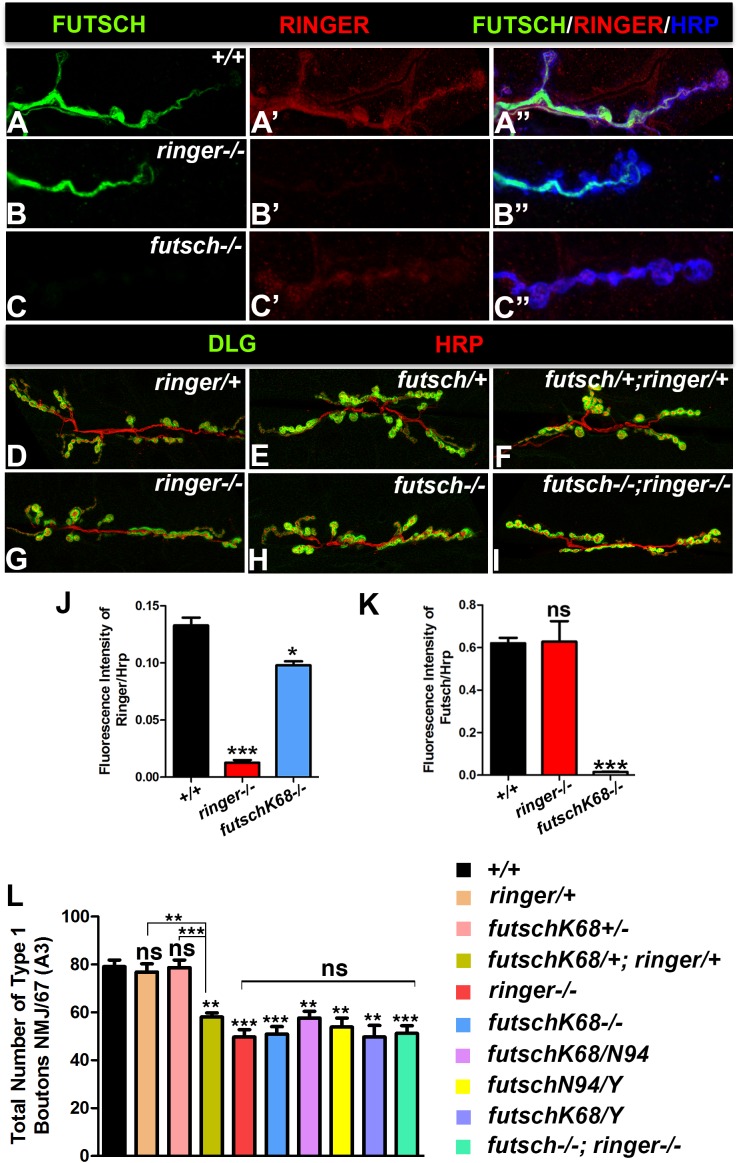
Ringer and Futsch localization and genetic interactions. **(A–C″)** Confocal images showing localization of Futsch (green, **A,A″,B,B″,C,C″**), Ringer (red, **A′,A″,B′,B″,C′,C″**) and Hrp (blue, **A″–C″**) at NMJ of wild type **(A–A″)**, *ringer–/–* **(B–B″)** and *futsch^K68/K68^* **(C–C″)**. Representative confocal images showing the synaptic growth in *ringer/+* **(D)**, *futsch^K68^/+* **(E)**, *futsch^K68^/+; ringer/+* **(F)**, *ringer–/–* **(G)**, *futsch^K68/K68^* **(H)**, and *futsch^K68/K68^; ringer–/–* **(I)** labeled with Dlg (green) and Hrp (red). Quantification of fluorescence intensity ratio of Ringer/Hrp **(J)** and Futsch/Hrp **(K)** and total Type 1 boutons **(L)** in specified genotypes. *n* = 15 animals per genotype, Error bars represent mean ± SEM [^∗^*p* ≤ 0.05, ^∗∗^*p* ≤ 0.01, ^∗∗∗^*p* < 0.001, not significant (ns)] (ANOVA).

Having established Ringer localization, phenotypic and functional consequences of its loss from the presynaptic NMJ terminals, we next set out to investigate what other protein/s Ringer might be functioning with in regulating MT cytoskeleton and synaptic growth. Vertebrate TPPPs and MAP1B and their respective *Drosophila* homologs, Ringer and Futsch, display phenotypic and functional similarities in neurite outgrowth and axonal MT organization (Hummel et al., 2000; Tokési et al., 2010; Aoki et al., 2014; Villarroel-Campos and Gonzalez-Billault, 2014; Mino et al., 2016; Szabó et al., 2017; Tymanskyj et al., 2017). More importantly, Futsch was also reported to regulate synaptic MT organization and growth (Hummel et al., 2000; Roos et al., 2000; Ruiz-Canada et al., 2004). We, therefore, wanted to examine whether Futsch could be a potential interacting partner of Ringer in regulating MT organization and synaptic growth. We first examined the localization of Ringer and Futsch at the wild type NMJ and in the mutant backgrounds of one another. We co-stained wild type third instar larvae to study the localization of Ringer (Figure 4A′,A″) with respect to Futsch (Figure 4A,A″) and the neuronal marker Hrp (Figure 4A″). Similar to Figure 1, here also we enhanced the brightness of the Ringer channel (red) uniformly across all genotypes (Figure 4A–C″) to assess the localization of Ringer with respect to Futsch. We found that Ringer (Figure 4A′) localized at low levels in similar compartment as Futsch (Figure 4A,A″) at the presynaptic NMJ terminals in wild type and, as expected, was absent in *ringer* null mutant larvae (Figure 4B′,B″). Despite lower levels of Ringer localization compared to Futsch in wild type NMJ terminals, we tested the extent of colocalization of Ringer and Futsch using spatial correlation analysis. An average of 80% colocalization of Ringer and Futsch was obtained using Manders correlation analyses (Manders et al., 1993) and ∼70% colocalization was observed using Pearson’s correlation analysis (Adler and Parmryd, 2010) (for details, see section “Materials and Methods”; Supplementary Figure S1). The regions of interests (ROIs) were selected from wild type NMJ branches from wild type larvae labeled with Ringer and Futsch similar to the ones shown in Figure 1A and Supplementary Figure S1. However, for colocalization analysis, images were not subjected to any modifications such as enhancement of brightness etc. for Ringer or Futsch. The spread of the data was plotted to see the distribution and extent of colocalization (Supplementary Figure S1). Based on these analyses, we conclude that Ringer, although expressed at much lower levels than Futsch, co-localizes with Futsch at the presynaptic NMJ terminals. Futsch localized to *ringer* mutant NMJs without showing any significant alterations in localization or levels (Figure 4B,B′). In *futsch* mutants, on the other hand, Ringer localization was very diffused at the NMJ (Figure 4C′,C″) when compared to wild type (Figure 4A′,A″). There was a significant reduction in levels of Ringer as quantified by ratio of fluorescence intensities of Ringer/Hrp at the *futsch* mutant larval NMJ (Figure 4J) compared to wild type. However, there was no significant difference in fluorescence intensity ratios of Futsch/Hrp in *ringer* mutants (Figure 4K) NMJ compared to the wild type. These data suggest that Ringer depends on Futsch for its proper localization at the presynaptic NMJ terminals.

Given the similar localization patterns of Ringer and Futsch at NMJ and a common synaptic undergrowth phenotype resulting from their individual loss of function (as seen in Figure 1 for *ringer* mutants; and as reported in Roos et al., 2000 for *futsch* mutants), we next wanted to study the consequences of the combined loss of Ringer and Futsch on synaptic growth (Figure 4D–I,L) in an attempt to establish if Ringer and Futsch display genetic interactions. We studied synaptic growth in a variety of genotypic combinations of *ringer* and/or *futsch*. Single heterozygotes of *ringer* (Figure 4D,L) and *futsch* (Figure 4E,L) displayed no significant difference in bouton growth when compared to wild type (Figure 4L). Double heterozygotes of *futsch/+;ringer/+* (Figure 4F,L), however, displayed a significant reduction in synaptic bouton growth compared to wild type and individual heterozygotes (Figure 4L) indicating that *futsch* and *ringer* display genetic interactions and may function together in regulating synaptic growth at the larval NMJ. Comparison of bouton counts in single mutants of *ringer* (Figure 4G,L) and *futsch* (Figure 4H,L) did not show any significant differences. Also, both male and female larvae of both *futsch* alleles analyzed, *futsch^K68^* and *futsch^N94^* as well as trans-allelic combination of *futsch^K68/N94^* showed similar reduction in bouton growth between one another as well as with respect to *ringer* mutants (Figure 4L). Double mutants of *futsch* and *ringer* (Figure 4I,L) also revealed bouton numbers that were not significantly different than *futsch* or *ringer* single mutants (Figure 4L). These data indicate that *futsch* and *ringer* display genetic interactions and are involved in promoting synaptic growth at the larval NMJ.

### Loss of Ringer and Futsch Lead to Decreased Levels of Acetylated- Tubulin at the NMJ

**FIGURE 5:**
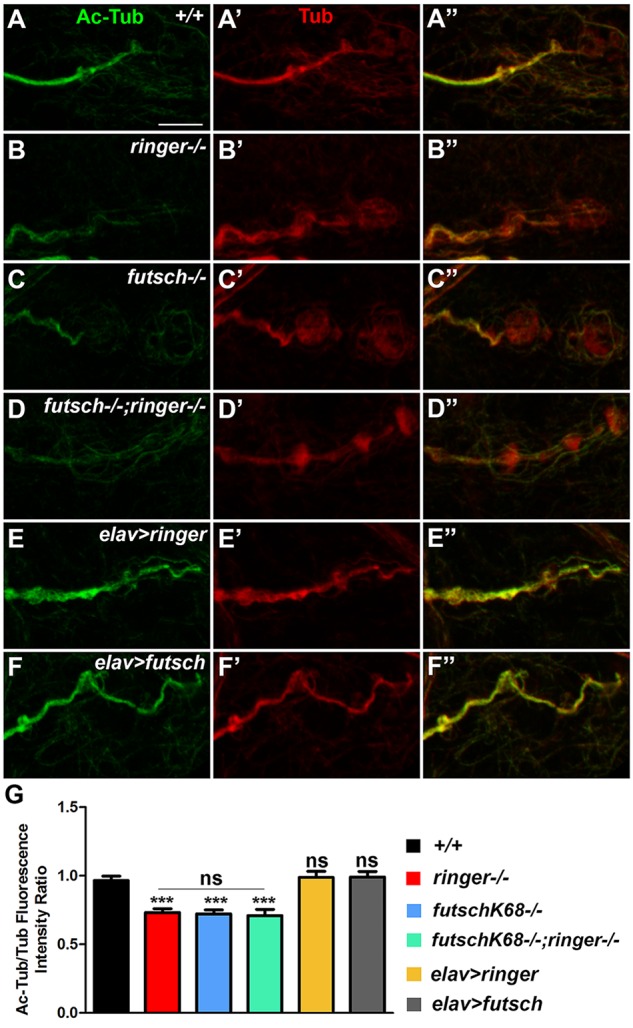
Reduced acetylated-tubulin levels in *ringer* and *futsch* mutant NMJs. **(A–F″)** Representative confocal images of NMJ branches showing Ac-Tub (green, **A–F″**) and Tub (red, **A′–F″**) localization in wild type **(A–A″)**, *ringer–/–* **(B–B″)**, *futsch^K68/K68^* **(C–C″)**, *futsch^K68/K68;^ringer–/–* **(D–D″)**, *elav-Gal4; UAS-ringer* **(E–E″)**, and *elav-Gal4/UAS-Futsch[EP1419]* **(F–F″)**. **(G)** Quantification of the ratio of Ac-Tub/Tub fluorescence intensities in represented genotypes. *n* = 10 animals (20 NMJ branches analyzed/genotype). Error bars represent mean ± SEM [^∗^*p* ≤ 0.05, ^∗∗^*p* ≤ 0.01, ^∗∗∗^*p* < 0.001, not significant (ns)] (ANOVA). Scale bar: 5 μm.

Given that TPPPs and MAPs are known to promote MT stability and post-translational modifications such as acetylation (Takemura et al., 1992; Conde and Caceres, 2009; Tokési et al., 2010; Szabó et al., 2017), we hypothesized that Ringer and Futsch might also be involved in regulating acetylation and stability of MTs. Since acetylation of α-tubulin at lysine 40 is a post-translational modification (Fukushima et al., 2009) that generally occurs on long-lived MTs, MT acetylation is viewed as a consequence of MT stability or age (Szyk et al., 2014). We stained larvae of various genotypic combinations (Figure 5) with Acetylated-Tub (Ac-Tub, green) and analyzed any changes with respect to total Tubulin (Tub, red). At the wild type NMJ, the Ac-Tub signal is intense within the synaptic core and shows a tight fasciculation of the proximal NMJ branch and is much fainter in the distal/terminal boutons (Figure 5A,A″; Nechipurenko and Broihier, 2012). Single mutants of *ringer* (Figure 5B–B″) and *futsch* (Figure 5C–C″) and their double mutants (Figure 5D–D″) showed a significant reduction in levels of Ac-Tub as quantified by ratio of fluorescence intensities of Ac-Tub/Tub compared to their wild type counterparts (Figure 5A–A″) (^∗∗∗^ significance of single and double mutants compared to wild type Figure 5G). There were no significant differences in fluorescence intensity ratios of Ac-Tub/Tub between individual single mutants and double mutants of *ringer* and *futsch* (ns- no significant difference between mutants, Figure 5G). We also analyzed the consequences of Ac-Tub localization and fluorescence levels following a presynaptic overexpression of full length Ringer and Futsch (using the Futsch[EP1419] line, Roos et al., 2000; Coyne et al., 2014) as seen in *elav-Gal4;UAS-ringer* (Figure 5E,E″,G) and *elav-Gal4/futsch[EP1419]* (Figure 5F,F″,G). Although ratio of the levels of Ac-Tub/Tub in Ringer overexpression (Figure 5E,E′,G) seemed elevated, it did not reach statistical significance compared to wild type (Figure 5A,A″,G). There was also no significant increase in ratio of levels of Ac-Tub in presynaptic Futsch overexpression (Figure 5F,F″,G). The total Tubulin levels analyzed in all of the specified genotypes did not show any changes at the NMJ synapses (Supplementary Figure S2). Together, these datasets revealed that individual and combined loss of Ringer and Futsch leads to significant decrease in Ac-Tub levels at the NMJ synapses.

### Ringer and Futsch Regulate MT Loop Organization at the NMJ

**FIGURE 6:**
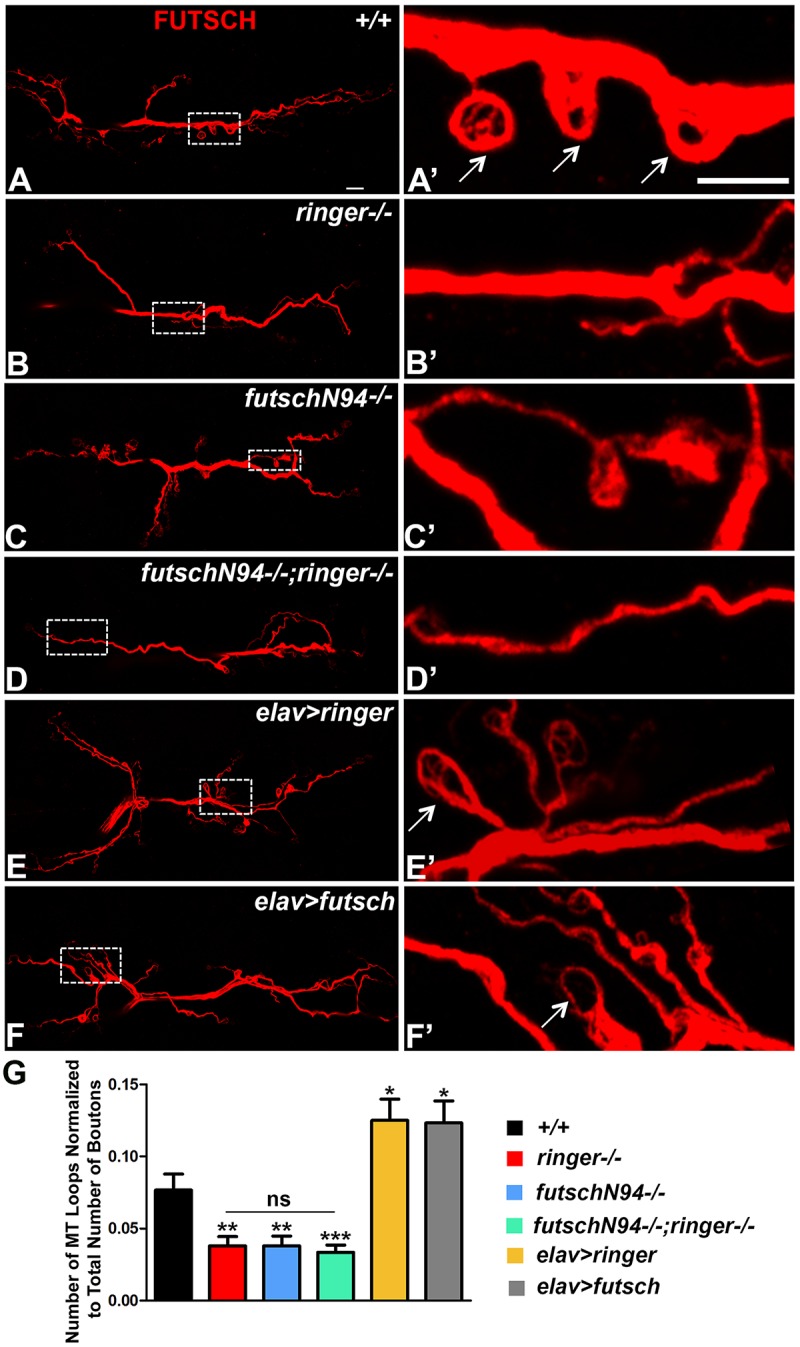
Ringer and Futsch regulate MT loops at NMJ. **(A–F)** Representative confocal images showing the entire NMJ arbor at low magnification stained with 22C10 in wild type **(A)**, *ringer–/–* **(B)**, *futsch^N94-/-^* **(C)**, *futsch^N94-/-^; ringer–/–* **(D)**, *elav-Gal4; UAS-ringer* **(E)**, and *elav-Gal4/UAS-Futsch[EP1419]* **(F)**. **(A′–F′)** Higher magnification of boxed regions from the corresponding lower magnification **(A–F)** is depicted in **(A′–F′)** of respective genotypes. Arrows **(A′,E′,F′)** highlight the MT loops. **(G)** Quantification of the number of MT loops/NMJ in represented genotypes. *n* = 15 animals of each genotype, Error bars represent mean ± SEM [^∗^*p* ≤ 0.05, ^∗∗^*p* ≤ 0.01, ^∗∗∗^*p* < 0.001, not significant (ns)] (ANOVA). Scale bars: **(A–F)** = 10 μM; **(A′–F′)** = 5 μM.

Since individual and combined loss of *ringer* and *futsch* showed reduced levels of Ac-Tub, we hypothesized that these mutants might also have a reduction in the number of MT loops at the NMJ. To this end, we studied NMJ MT loops by staining larvae of various genotypes with anti-Futsch (Figure 6). Loops represent a normal feature of MT sub-synaptic architecture and are typically present in low numbers at wild type *Drosophila* NMJs mostly at branch points and within terminal boutons (Roos et al., 2000; Ruiz-Cañada and Budnik, 2006; Miech et al., 2008; Nechipurenko and Broihier, 2012). A small number of MT loops also appear periodically along the nerve terminal (arrows, Figure 6A′). A rearrangement of MT-loop architecture may occur during bouton division as well. Futsch, in particular, is necessary for this process and identifies cytoskeletal loops that traverse the lateral margin of select synaptic boutons (Roos et al., 2000). Tubulin loops have also been described in other systems, in which they are proposed to highlight stable MTs (Fritsche et al., 1999; Bergstrom et al., 2007; Hendricks and Jesuthasan, 2009; Mino et al., 2016).

As expected, single mutants of *ringer* (Figure 6B,B′,G) and *futsch* (Figure 6C,C′,G) as well as the double mutants (Figure 6D,D′,G) displayed a significant reduction in the total number of MT loops as assayed by anti-Futsch compared to wild type (Figure 6A,A′,G). Interestingly, presynaptic overexpression of Ringer (Figure 6E,E′,G) and Futsch (Figure 6F,F′,G) showed a significant increase in the number of NMJ MT loops. Higher magnifications of the boxed regions in Figure 6A–F corresponding to specified genotypes are presented in panels Figure 6A′–F′ . There was no significant difference in the unbundled MTs at the NMJ across all of the specified genotypes (Supplementary Figure S3). Together with the Ac-Tub localization and levels (Figure 5), the findings on NMJ MT loops further demonstrate that Ringer and Futsch function to promote synaptic MT stability and organization.

### Ringer, Futsch, and Tubulin Exist as a Biochemical Complex

**FIGURE 7:**
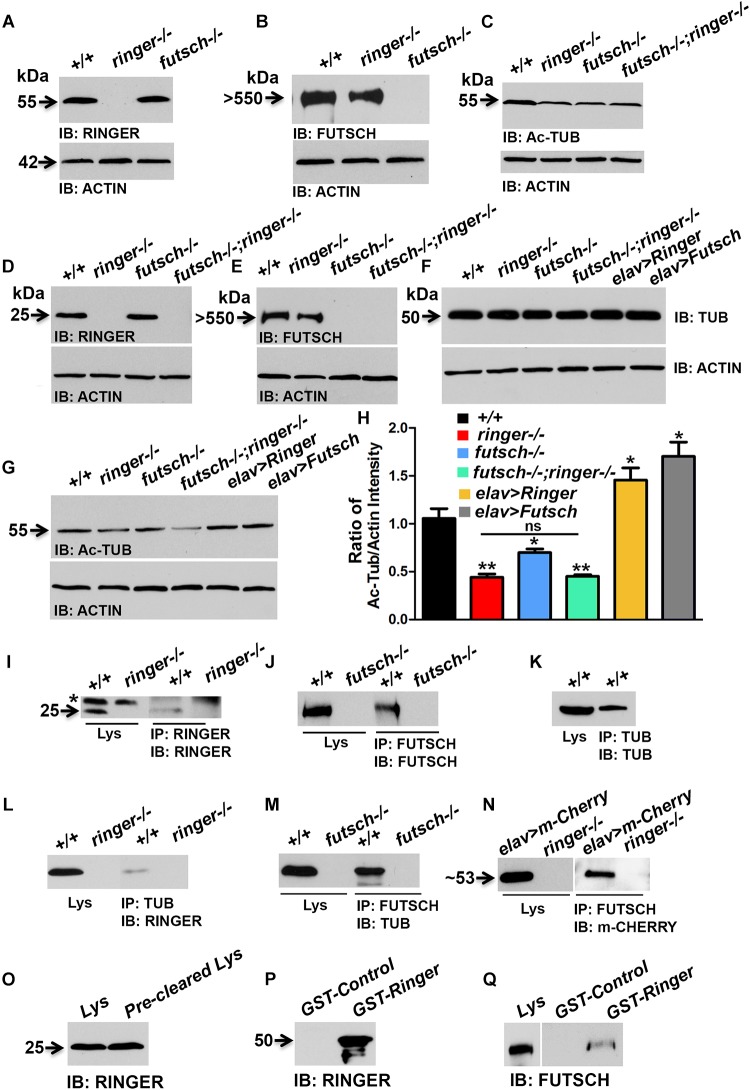
Biochemical interactions between Ringer and Futsch. **(A–G)** Representative immunoblots from larval lysates **(A–C)** and fly head lysates **(D–G)** showing total levels of Ringer **(A,D)**, Futsch **(B,E)**, Ac-Tub **(C,G)**, and Tub **(F)** of specified genotypes. Actin **(A–G)** was used as the loading control. **(H)** Quantification of the ratio of band intensities of Ac-Tub/Actin in represented genotypes. Adult head lysates of wild type (+/+) and *ringer–/–* probed with anti-Ringer **(I)**; wild type and *futsch–/–* probed with anti-Futsch **(J)** and wild type probed with anti-Tub **(K)** antibodies. Head lysates of wild type in combination with *ringer–/–* **(I)** and *futsch–/–* **(J)** immunoprecipitated with anti-Ringer and anti-Futsch, respectively. There is presence of a non-specific band (asterisk, **I**) in wild type and mutant lysate and IP lanes when probed with rat anti-Ringer antibodies. Wild type head lysates immunoprecipitated with anti-Tub **(K)**. **(L–N)** Head lysates of wild type (+/+) in combination with *ringer–/–* **(L)** and *futsch–/–* **(M)** co-immunoprecipitated with anti-Tub and anti-Futsch, respectively and probed with anti-Ringer **(L)** and anti-Tub **(M)**. Head lysates from *elav-Gal4;UAS-m-Cherry-Ringer*, and *ringer–/–* probed with anti-m-Cherry **(N)** and co-immunoprecipitation of lysates from *elav-Gal4;UAS-m-Cherry-Ringer*, and *ringer–/–* probed with anti-m-Cherry **(N)**. **(O–Q)** Adult wild type head lysates probed with anti-Ringer **(O)** and anti-Futsch **(Q)**. Immunoblots of GST-Control and GST-Ringer probed with anti-Ringer **(P)** and anti-Futsch **(Q)**. Note that blots separated by a space **(N,Q)** were probed separately. *futsch^K68/K68^* mutants were used for all biochemical analyses. *n* = 3 (each experiment was done independently three times and the most representative blots are shown).

Given that Ringer localization was found diffused in *futsch* mutants (Figure 4) and levels of Ac-Tub and MT loops were significantly reduced in individual and combined loss of *ringer* and *futsch* mutant NMJ (Figure 5, 6, respectively), we wanted to examine the total levels of these proteins in Ringer and Futsch loss- and gain-of-function. Also, given that vertebrate TPPPs bind and polymerize tubulin and stabilize MTs (Hlavanda et al., 2002), we wanted to examine if the *Drosophila* TPPP/Ringmaker and MAP1B/Futsch exist as an *in vivo* biochemical complex together with Tub. To address these questions, we performed immunoblots, immunoprecipitations (IPs) and GST pull-down assays. Immunoblots were performed both from third instar larvae (Figure 7A–C and Supplementary Figure S4) and adult fly heads (Figure 7D–G) and the results from both tissue types were found to be similar for the proteins analyzed. The remainder biochemical analyses were therefore performed with fly head lysates due to the ease of handling and availability in larger quantities for IP and GST pull-down assays.

Immunoblot analysis showed that Ringer (Figure 7A,D) and Futsch (Figure 7B,E) levels were not significantly affected in each other’s mutant backgrounds when compared to wild type. Total Tub levels were also unaltered in single and combined loss of *ringer* and *futsch* as well as their presynaptic overexpression (Figure 7F). However, consistent with the findings of the Ac-Tub fluorescence levels at NMJ (Figure 5), total levels of Ac-Tub from lysates of single and double mutants of *ringer* and *futsch* was also found to be significantly decreased when compared to wild type (Figure 7C,G,H). Ac-Tub levels were significantly elevated in presynaptic Ringer and Futsch overexpression compared to wild type (Figure 7G,H). Actin was used as loading control for all immunoblots (Figure 7A–G).

Immunoprecipitations using Ringer (Figure 7I) and Futsch (Figure 7J) efficiently precipitated Ringer and Futsch, respectively, from wild type lysates that were missing from their respective mutant lysates thus confirming the specificity of the IPs. A non-specific band (asterisk, Figure 7I) was detected in the wild type and mutant lysate and IP lanes, when probed with rat anti-Ringer antibodies. Similarly, IP using Tub could also precipitate Tub from wild type lysate (Figure 7K). Co-IPs with Tub (Figure 7L) and Futsch (Figure 7M) could detect Ringer and Tub, respectively, suggesting that Ringer and Futsch associate with Tub in an *in vivo* complex. No association with Futsch and Ringer was observed with IPs using antibodies to endogenous protein at normal levels, thus, overexpression and GST pull down was necessary to establish their association in a complex. We first overexpressed *UAS-m-Cherry Ringer* using *elav-Gal4* and performed co-IP using anti-Futsch (Figure 7N) and could successfully detect m-Cherry Ringer. In order to further consolidate the possible interactions between Futsch and Ringer, we utilized previously generated GST-Ringer fusion protein (Mino et al., 2016). GST-Ringer was incubated with wild type fly head lysates that contain endogenous Ringer (Figure 7O) and Futsch (Lys, Figure 7Q) and pulled-down by Glutathione-Sepharose beads. We first confirmed the specificity of the pull-down experiment by probing GST-Ringer and GST-Control with anti-Ringer (Figure 7P) and anti-GST (Supplementary Figure S4) antibodies. We detected Ringer in the GST-Ringer lane (∼50 kDa, Figure 7P) that was absent in the GST-Control lane. Similarly, we could detect the presence of GST in GST-Control (26 kDa) and GST-Ringer (∼50 kDa) lanes in a Coomassie stained gel and when probed with anti-GST antibodies (Supplementary Figure S4). A significant level of Futsch was detected in the GST-Ringer lane that was not present in the GST control lane (Figure 7Q). These results further demonstrate that Ringer and Futsch exits *in vivo* as a molecular complex and function together to maintain MT stability.

## Discussion

Polymerization of MT involves a variety of proteins such as the well-known MAPs and a comparatively lesser known, yet highly significant, TPPPs. Our studies reported here identify the *Drosophila* TPPP/Ringer in regulating presynaptic MT organization, stability and synaptic growth in concert with the *Drosophila* MAP1B/Futsch. Based on our findings, we propose that Ringer and Futsch function in a complex that is essential for proper presynaptic MT organization and synaptic growth and opens up the prospect of future genetic investigations into the regulation of MT dynamics using *Drosophila.*

### Ringer in NMJ Synapse Development and Function

While regulation of synaptic MTs and the range of proteins that affect synaptic MT organization and function are not well-characterized, synaptic MTs have been implicated in regulating synaptic bouton growth (Roos et al., 2000). Thus, understanding the regulation of MT assembly, organization and dynamics in synaptic terminals is crucial for understanding synapse development and function. Our findings demonstrate that loss of Ringer affects synaptic bouton growth at the NMJ (Figure 1). The growth of NMJ synapses in *Drosophila* has been postulated to occur either through a process called intercalation where existing synaptic boutons space apart with new boutons inserted between them, or by end addition where new boutons are added at the ends of existing string of boutons (Zito et al., 1999). Synapse growth is also thought to occur from budding of existing boutons (Zito et al., 1999). While future studies will determine which of these processes may be compromised in *ringer* mutants leading to a reduction in the number of NMJ synaptic boutons, Ringer can be added to the increasing repertoire of proteins involved in the modulation of synaptic growth. It is likely that the regulation of the cytoskeleton by Ringer may have a profound impact on the balance between synaptic growth and stability. Since synaptic growth can be under both positive and negative regulations, one issue of interest would be to determine what genes are upstream and downstream of Ringer and define a signaling cascade that modulate synaptic growth.

The observations that the apposition of the presynaptic AZ protein, BRP, with the GluR receptor fields were not severely disrupted in *ringer* mutants (Figure 1) suggest that Ringer might not be crucial for proper placement of pre- and post-synaptic specializations at the synaptic boutons. Interestingly, number of BRP-positive puncta/bouton area was significantly increased in *ringer* mutants than wild type (Figure 1). The synaptic ultrastructure of *ringer* mutants also revealed an increase in AZ number as well as disrupted AZ morphology (Figure 2). Thus, one possibility is that Ringer may directly play a role in AZ organization by interacting with BRP or indirectly through other proteins. It is also possible that disorganized MTs due to loss of Ringer may simply impact the proper assembly of AZs in the synaptic boutons. While elucidating the role of Ringer in AZ organization is an interesting topic of future research, it is important to note that this role of Ringer may or may not be dependent on Futsch. Recent findings report that *futsch* mutants, contrary to *ringer* mutants, have a decrease in AZ number and density at the larval NMJs but normal AZ ultrastructural morphology (Lepicard et al., 2014) further underscoring the fact that these proteins may coordinate unique axonal cytoskeletal functions during synapse organization.

*ringer* mutants showed a decrease in bouton numbers but an increase in AZs/bouton area as revealed both by Brp immunostaining (Figure 1) and EM analyses (Figure 2). This phenotype could result in unchanged spontaneous firing of the minis as is reflected from no significant changes in mEJP frequency (Figure 3). At the same time the evoked EJP amplitude was decreased in *ringer* mutants. The increase in AZ numbers did not translate directly into increased miniature frequency, as loss of Ringer may also affect synapse ultrastructure that could still be abnormal at the more molecular level. Analysis of the synaptic vesicles (clustered and docked) at the AZs in the presynaptic terminals of *ringer* mutants also did not reveal any significant differences compared to controls. It is quite likely that significant decrease in EJP amplitude and quantal content might reflect a lower release probability and possibly defects in the synaptic release machinery. Altogether, Ringer loss reflects a presynaptic defect in neurotransmission machinery.

### Ringer Localization and Its Role in Pre-synaptic MT Organization

Both our present and previously published studies on Ringer (Mino et al., 2016) reveal an interesting spatio-temporal pattern and differential levels of Ringer localization in the *Drosophila* nervous system during development. Ringer displayed a temporally dynamic expression in neurons during early embryonic stages followed by an expression at the midline glia during later stages of embryonic ventral nerve cord development (Mino et al., 2016). Interestingly, in vertebrates, TPPP is predominantly expressed in the CNS oligodendrocytes and plays a critical role in myelin maturation (Skjoerringe et al., 2006; Lehotzky et al., 2010; Ota et al., 2014). Given Ringer’s localization in both neuronal and glial cell types in the *Drosophila* embryonic CNS, it is possible that mammalian TPPP may also be expressed at lower/undetectable levels in neurons in physiological conditions. Under pathological conditions though, TPPPs are reported to be enriched and colocalize with α-Synuclein in neuronal and oligodendroglial inclusions that are characteristic of Synucleinopathies (Kovács et al., 2004). Ringer also has differential levels of wild type localization in third instar larvae as it is expressed at higher levels in larval axons (Mino et al., 2016) but at much lower levels at the presynaptic NMJ terminals (this study). The NMJ localization is mostly cytoplasmic but also seems to associate with Futsch, which localizes at higher levels to the core MT cytoskeleton (Figure 4 and Supplementary Figure S1).

MT assembly and dynamics are regulated by several factors and mechanisms, such as MT-assembly promoting factors, MT stabilizing/destabilizing factors, MT severing proteins and MT post-translational modifications that affect MT stability (Conde and Caceres, 2009; Janke and Kneussel, 2010). As cells respond to physiological needs, they constantly adapt their MT arrays by modulating the balance between dynamic and stable MT subpopulations (Szyk et al., 2014). This is also achieved through acetylation which occurs primarily on MTs and can be abundant on long lived stable MTs. Our studies revealed that Ringer together with Futsch regulates levels of Ac-Tub at the NMJ with single and double mutants displaying significantly decreased levels of acetylation (Figure 5). These *in vivo* findings are in line with previously reported cell culture data showing down regulation of TPPP by specific si-RNA resulted in decrease of Ac-Tub levels (Tokési et al., 2010). The control of acetylation level of MT network is an important factor for the regulation of MT architecture and maintenance of its integrity (Li and Yang, 2015). Our data suggest that one of the aspects of Ringer functions would thus be to regulate the MT architecture possibly by regulating levels of MT acetylation.

The stabilization of MTs during neuronal maturation also underlies axonal specification and growth. Data from *Drosophila* have shown that the conversion of a motile growth cone into a presynaptic terminal is associated with the appearance of a hairpin MT loop in the growth cone (Roos et al., 2000). Homozygous mutations in both *ringer* and *futsch* alter MT loop formation, a process that has been implicated as a phenomenon reflective of MT stability and budding of new boutons (Roos et al., 2000; Ruiz-Cañada and Budnik, 2006; Nechipurenko and Broihier, 2012). While individual and combined loss of *ringer* and *futsch* resulted in reduced levels of synaptic Ac-Tub (Figure 5) and reduction in NMJ MT loops (Figure 6), overexpression of Ringer and Futsch showed the opposite (Figure 6). These findings are consistent with the *in vitro* cell culture experiments and biochemical Tubulin assays that showed that Ringer affects MT polymerization; with Ringer-expressing cells forming a circular ring instead of regularly distributed MTs (Mino et al., 2016). Vertebrate MAP1B may also be involved in MT loop formation as revealed by *in vitro* overexpression of MAP1B (Tögel et al., 1998).

There is also a group of MT-severing proteins that regulate synaptic MT stability and growth at the NMJ. These are Spastin (Sherwood et al., 2004) and Katanin 60 (Mao et al., 2014). Spastin is enriched in axons and is highly abundant in presynaptic terminals. Knockdown of Spastin causes a severe reduction in synaptic arbor and an increase in stable and looped MTs at synaptic terminals (Sherwood et al., 2004). Similarly, loss of Katanin 60 also resulted in increased MT loops and levels of Ac-Tub (Mao et al., 2014) suggesting that these protein functions are contrasting to that of Ringer and Futsch. Vertebrate Spastin is critically required for axonal outgrowth during zebrafish embryonic development (Wood et al., 2006). Also, axon branch loss at the developing mouse NMJ is mediated by branch-specific MT severing by Spastin, which results in local disassembly of the MT cytoskeleton with subsequent dismantling of branches (Brill et al., 2016). Mutations in Spastin have also been associated with increased stabilization of MT network (Evans et al., 2005). Recently, it has also been shown that in HeLa cells, the two isoforms of Spastin harboring a missense mutation increases the levels of Ac-Tub (Plaud et al., 2018). Thus, the broader implications from all of these findings could be that a fine balance of acetylation/de-acetylation kinetics may underlie proper MT organization and synaptogenesis.

The primary intracellular target of TPPP is tubulin/MT under both *in vitro* and *in vivo* conditions and displays extensive MT bundling activity (Hlavanda et al., 2002; Mino et al., 2016). One of the crucial factors affecting the function of MT network is its acetylation by the action of acetyltransferase complex (Ohkawa et al., 2008) as well as histone deacetylase 6 (HDAC6; Hubbert et al., 2002) and Sirtuin-2 (SIRT2; North et al., 2003). *In vitro* studies suggest that mammalian TPPP modulates MT acetylation by binding to HDAC6 and inhibits its activity, resulting in a reciprocal increase in MT acetylation (Tokési et al., 2010). HDAC6 is commonly considered to be a tubulin-deacetylase because chemical inhibition of this enzyme significantly increases MT acetylation in neurons (Hubbert et al., 2002). Similar to HDAC6, a more recent study showed the tubulin deacetylase (SIRT2) to play a role with TPPP in regulating MT dynamics and stability (Szabó et al., 2017). Thus, TPPP-directed deacetylase inhibition can be speculated as one of the mechanisms for the fine control of the dynamics and stability of the MT network. It will be interesting to further investigate whether *Drosophila* Ringer and/or Futsch may form a larger molecular complex that involves aspects of HDAC6 and SIRT2 in regulating MT dynamics and potentially synaptic growth at the NMJs. *In vitro* studies have also demonstrated that TPPP influences MT dynamics by decreasing the growth velocity of MT plus ends (Tokési et al., 2010). While our future studies will investigate how *Drosophila* Ringer modulates the dynamics and stability of the MT network, one can speculate based on the findings from the vertebrate TPPP, that these mechanisms could involve its MT assembly promoting, cross-linking and/or acetylation enhancing activities.

### Protein–Protein Interactions of Ringer/TPPPs

The biochemical analyses of Ringer reported here provide important insights into its role in regulating the MT cytoskeleton. It is interesting that the overall levels of Ringer did not change in *futsch* mutants compared to the control. This finding was consistent whether the total Ringer levels were assayed from larval tissues or adult head lysates. However, while the total Ringer levels were unchanged, the synaptic Ringer localization displayed a significant alteration compared to control (Figure 4) raising the possibilities that, in the absence of Futsch, either Ringer levels significantly decreases in the presynaptic terminals or Ringer just fails to localize in its proper place and instead gets diffuse. However, total Tub levels and that of Ac-Tub (Figure 7) were consistent with what was observed at the synapses. Irrespective of tissue type, our findings reveal a remarkable consistency in demonstrating that Ringer and Futsch regulate synaptic and overall MT stability: (1) Ac-Tub levels in synapses (Figure 5), (2) synaptic MT loops (Figure 6) and (3) total Ac-Tub levels (Figure 7), each of these parameters were found to be affected similarly with a reduction in individual and combined loss of *ringer* and *futsch* and an elevation in their respective overexpression.

Although not in the context of intercellular protein–protein interactions in the synapses, there are reports of some TPPP interacting proteins (Supplementary Table S1). Consistent with published reports (Hlavanda et al., 2002), Ringer being a Tub-binding protein was further reiterated by their presence in the IP complex. As expected, the MAP1B/Futsch also existed in a complex with Tub. Interestingly, while we could not detect endogenous Ringer and Futsch in the same IP complex, we could detect m-Cherry tagged Ringer from an overexpression experimental paradigm. These datasets are reflective of an inability of the endogenous proteins to be detected either due to: (1) a huge difference in their molecular weights (Ringer being ∼25 kDa and Futsch over 550 kDa); (2) the relative abundance of the endogenous proteins; and (3) the binding affinity or the stoichiometry of the complex. However, the GST pull-down assays further established Futsch as an interacting partner of Ringer. Having established Ringer and Futsch as a complex, it will be interesting to investigate what other known as well as yet to be identified proteins will likely be recruited to this complex. Moreover, the large size and multiple domains of Futsch alone may allow it to complex with several others in the presynaptic terminals. An issue of interest, then, will be to determine how these complexes are assembled together with the variety of interactions with the post-synaptic targets. Also interesting will be to see if these protein–protein interactions are conserved across species, particularly in vertebrates and what role they will play in regulating MT dynamics. Together our results reveal that changes in MT organization are an essential aspect of synapse development and function and Ringer, a member of the unique and highly conserved TPPP family of proteins, plays a role in regulating MT stability and synaptic organization.

## Materials and Methods

### *Drosophila* Stocks

The *Drosophila* strains used in this study were isogenized *w^1118^ Canton-S* line (a gift from V. Budnik) used as the wild type control, *ringer^915^, UAS-Ringer* and *UAS-m-Cherry Ringer* (Mino et al., 2016). All other fly stocks, including *futsch^K68^, futsch^N94^, futsch[EP1419]* were obtained from Bloomington Stock Center, Indiana. Flies were maintained at 22°C, 50% humidity and with a 12-h light/dark cycle. To avoid over-crowding, all fly lines for various phenotypic analyses were set up using 10 females and 5 males and transferred every 24 h into fresh media.

### Immunohistochemistry and Confocal Imaging

Wandering third-instar larvae of both sexes from various genotypes were dissected and fixed in Bouin’s fixative for 15 min and processed for immunohistochemistry as previously described (Chen et al., 2012). Confocal images of all genotypes of larvae belonging to the same experimental group were acquired using the same settings with a Zeiss LSM710 confocal microscope and image editing was done using Adobe Photoshop. Primary antibodies used were FITC-conjugated anti-HRP (1:250, Jackson ImmunoResearch Laboratories), guinea pig anti-Ringer (1:250, Mino et al., 2016), anti-Ac-Tub (1:1000, T7451, Sigma), anti-Tubulin (1:1000, 2144S, Cell Signaling), and mouse monoclonal anti-Dlg (1:1000, 4F3), anti-Brp (1:250; NC82), anti-Futsch (1:1000; 22C10), and anti-GluR IIA (1:250, 8B4D2) were obtained from Developmental Studies Hybridoma Bank (DSHB), University of Iowa. Secondary antibodies conjugated to Alexa 488 and 568 (Invitrogen-Molecular Probes) were used at 1:400 dilution.

### Colocalization Analysis

Colocalization of Ringer and Futsch were analyzed from confocal images using “coloc2” plugin in FIJI software (Schindelin et al., 2012). Amongst the different statistical tests provided by this plugin, Pearson’s and Manders method (Manders et al., 1993; Adler and Parmryd, 2010; Dunn et al., 2011) calculates the colocalization coefficient of fluorescent intensities in two different channels ranging from 0 to 1 (0: no colocalization and 1: high colocalization) This plugin also provides the choice to use Costes method (Costes et al., 2004) to automatically subtract threshold value that considers the background of the pixels intensities. We performed coloc2 analysis by specifying ROI at the NMJ branches and quantified the colocalization of Ringer with Futsch.

*n* = 5 animals of wild type.

### Immunoblotting and Immunoprecipitations

Adult fly heads and 3^rd^ instar larval musculature without brain lobes, ventral nerve cord or any attached imaginal disks were homogenized in ice-cold lysis buffer (Banerjee et al., 2017). The supernatants with equal amounts of proteins from each genotype were separated on SDS-PAGE for immunoblotting with respective antibodies. For immunoprecipitations (IP) studies, fly heads were processed according to previously described protocols (Banerjee et al., 2006, 2017). Each experiment was done independently three times and the most representative blots are shown. Primary antibodies used for immunoblotting were guinea pig anti-Ringer (1:15,000), rat anti-Ringer (1:1000), anti-Futsch (1:2000), anti-Ac-Tub (1:5000, Sigma), anti-m-Cherry (1:15,000; Novus Biologicals), anti-Tubulin (1:5,000; Cell Signaling) and anti-β Actin (1:15,000, 4967S, Cell Signaling).

### GST Pull-Down Assay

For GST pull-down assay, fly head lysates from wild type were prepared similar to the IP experiments. GST and GST-Ringer fusion proteins were prepared as previously described (Mino et al., 2016). Fly head lysates were incubated with 10 μg of GST and GST-Ringer, respectively, for 16 h at 4°C to maximize the binding, then incubated with Glutathione-Sepharose beads (Sigma) for another 1 h at 4°C, followed by three washes with lysis buffer, twice with PBST and twice with PBS. The pull-down proteins were then eluted with reduced Laemmli sample buffer before being analyzed by SDS-PAGE and immunoblotting.

### Quantification and Statistical Analysis

Bouton number quantifications (*n* = number of larvae analyzed) were performed from muscles 6/7 of abdominal segment 3 (A3) by staining of the body wall muscle preparations with anti-HRP and anti-Dlg.

Fluorescence intensity measurements for Ac-Tub were quantified as previously reported (Banerjee et al., 2017) using Image J (NIH, United States) from confocal slices of Z-stack images compressed using maximum projection functions, which were stained in combination with anti-Tub antibodies. We used NMJ branches with bouton clusters for our analysis. Regions of interest were selected by outlining the Tub staining of a given NMJ branch and the same ROI was used for the Ac-Tub channel for assessment and quantification of fluorescence intensity. Mean signal intensity for Ac-Tub antibody was calculated and normalized to the mean Tub intensity for each NMJ branch. 20 NMJ branches from 10 larvae were analyzed for various genotypes in Figure 5. All genotypes listed under the same quantification groups were stained, processed for imaging and quantified under identical parameters and settings.

Fluorescence intensity measurements and quantification of Ringer/Hrp and Futsch/Hrp (Figure 4) were done similar to Ac-Tub/Tub. Regions of interest were selected by outlining the Hrp staining of a given NMJ branch and the same ROI was used for either the Ringer or Futsch channel for assessment and quantification of fluorescence intensity.

NMJ boutons from various genotypes were stained with anti-BRP for labeling the active zones and were analyzed on FIJI using the ROI plugin. Each BRP puncta circumference was selected manually and added as a new ROI. All ROIs were then measured automatically using the plugin. Color thresholds were kept constant in all analyzed images across genotypes.

Image J (NIH, United States) was used for quantification of band intensities of various proteins with respect to their loading control for the immunoblot experiments. A ratio of the intensities of the bands of interest with their respective Actin loading control were used for statistical analysis from three independent experiments.

All statistical analyses were performed using the GraphPad PRISM software and data are presented as mean ± SEM. Statistical significance was determined by one way ANOVA followed by *post hoc* Tukey’s multiple comparison test and Student’s *t*-test. Error bars represent mean ± SEM (^∗∗∗^*p* < 0.001; ^∗∗^*p* ≤ 0.01; ^∗^*p* ≤ 0.05; ns, not significant).

For all quantification, the statistical significance immediately above the bars is with respect to the control genotype for that experimental group.

### Electron Microscopy and Morphometric Analysis

Ultrastructural analyses of third-instar larval NMJs were processed for TEM as previously described (Banerjee et al., 2017). Briefly, third-instar larval filets were dissected in ice-cold, Jan’s 0.1 mM Ca^2+^ saline, pH 7.2, and fixed in 4% paraformaldehyde/1% glutaraldehyde in 0.1 M cacodylic acid, pH 7.2 for 30 min at room temperature followed by overnight fixation at 4°C. The fixed filets were rinsed in 0.1 M cacodylic acid, pH 7.2 and post-fixed in 2% aqueous osmium tetroxide for 1 h, followed by rinsing and dehydration in increasing ethanol concentration. Samples were incubated for an hour in propylene oxide and gradually infiltrated in increasing resin to propylene oxide ratio (1:2, overnight; 2:1, 6 h; and full resin for 36 h with constant agitation). Samples were embedded in flat silicone molds with Polybed resin and cured in the oven at 55°C for 36 h.

5 larvae were processed for EM analysis from each of the genotypes shown in Figure 2. The number of boutons (n) analyzed for each genotype was 50. Image J was used for morphometric analysis of EM images of only Type 1b boutons from segments A2 and A3 as previously described (Li et al., 2007; Chen et al., 2012).

For the quantification of clustered vesicles, we included vesicles located within the range of 250 nm away from a T-bar (AZ), as performed previously (Giagtzoglou et al., 2009). For the quantification of docked vesicles, we included vesicles that surround the T-bar (250 nm) and are 30 nm away from the plasma membrane.

### Electrophysiology

Electrophysiological analysis of larval NMJ was performed as previously described (Chen et al., 2012). All recording were made on muscle 6, A3 of third-instar larvae. All data were collected only when resting membrane potential was below -65 mV. Only one muscle per larva was recorded for each experiment. Excitatory junction potentials (EJPs) were evoked at a fixed duration of 0.3 ms at 0.2 Hz of stimuli rate, through a glass capillary electrode (internal diameter, ∼10 μM). The applied currents were 6 + 3 μA. Twenty evoked EJPs were recorded for each muscle for analysis. Miniature EJP (mEJP) events were collected for 2 min. Both EJPs and mEJPs were amplified with an Axoclamp 900A, under the control of Clampex 10 (Molecular Devices). All experiments were performed at 20–22°C. EJPs were analyzed with Clamfit 10.4 software (Molecular Devices). mEJPs was analyzed using the Mini Analysis Program (Synaptosoft). Evoked EJP amplitude was corrected by using non-linear summation (Feeney et al., 1998). The quantal content was calculated from individual muscles by ratio of the averaged EJP and averaged mEJP amplitude. Statistical analyses of EJP and mEJPs between genotypes were done using Student’s *t*-test (Sigma- Plot 10.0, Systat Software). Error bars represent mean ± SEM (^∗∗∗^*p* < 0.001; ^∗∗^*p* ≤ 0.01; ^∗^*p* ≤ 0.05; ns, not significant).

## Author Contributions

QS, AS, and JX performed the experiments and analyzed data. YL performed the electrophysiology experiments. YL and GN analyzed the data. SB conceptualized and designed the research, performed the experiments, analyzed the data, and wrote the manuscript.

## Conflict of Interest Statement

The authors declare that the research was conducted in the absence of any commercial or financial relationships that could be construed as a potential conflict of interest.
