# Supplementary material for: Tubulin Polymerization Promoting Protein, Ringmaker, and MAP1B Homolog Futsch Coordinate Microtubule Organization and Synaptic Growth
Source: Front Cell Neurosci. 2019 May 15;13:192. doi: 10.3389/fncel.2019.00192 (PMC6529516; doi:10.3389/fncel.2019.00192)
Supplement: Supplementary file 1 [file Data_Sheet_1.pdf]

Supplementary Figure S1

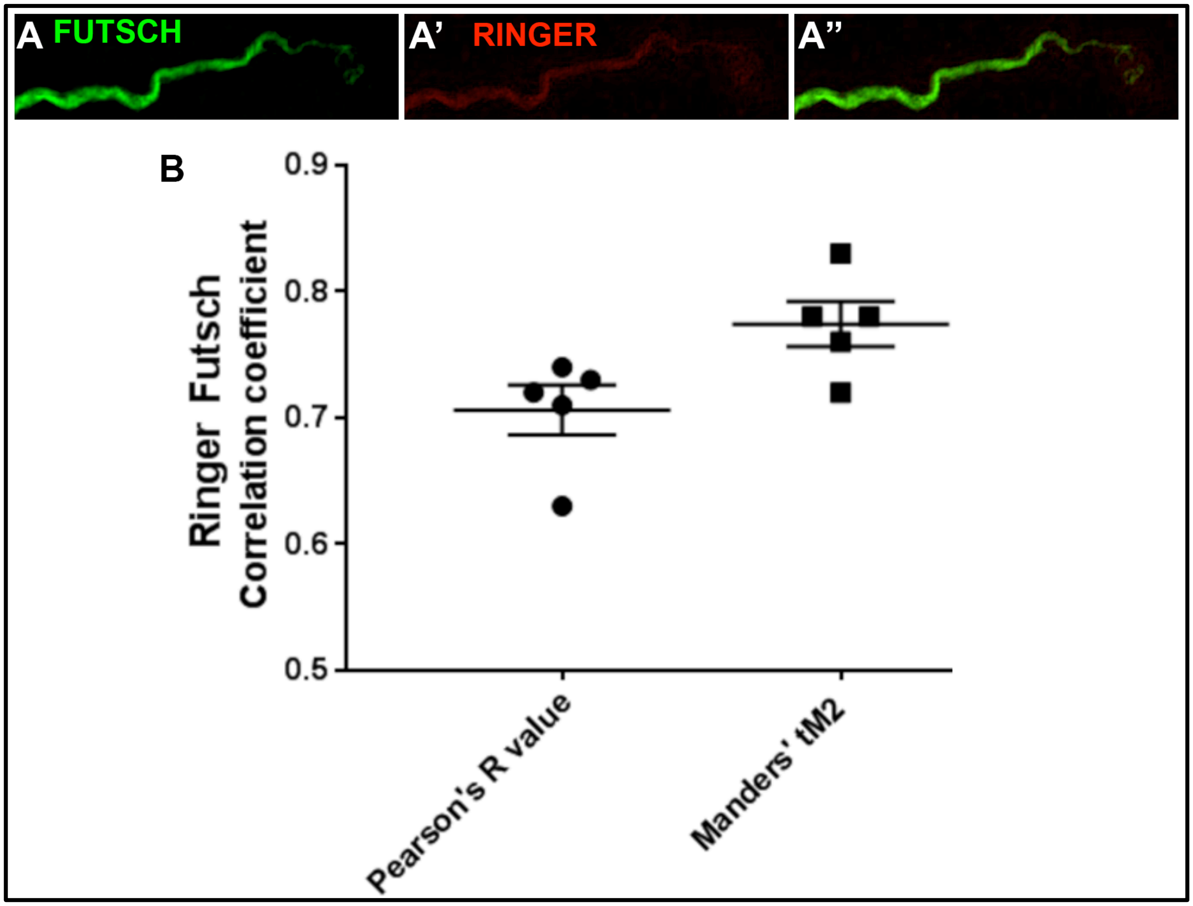

Supplementary Figure S2

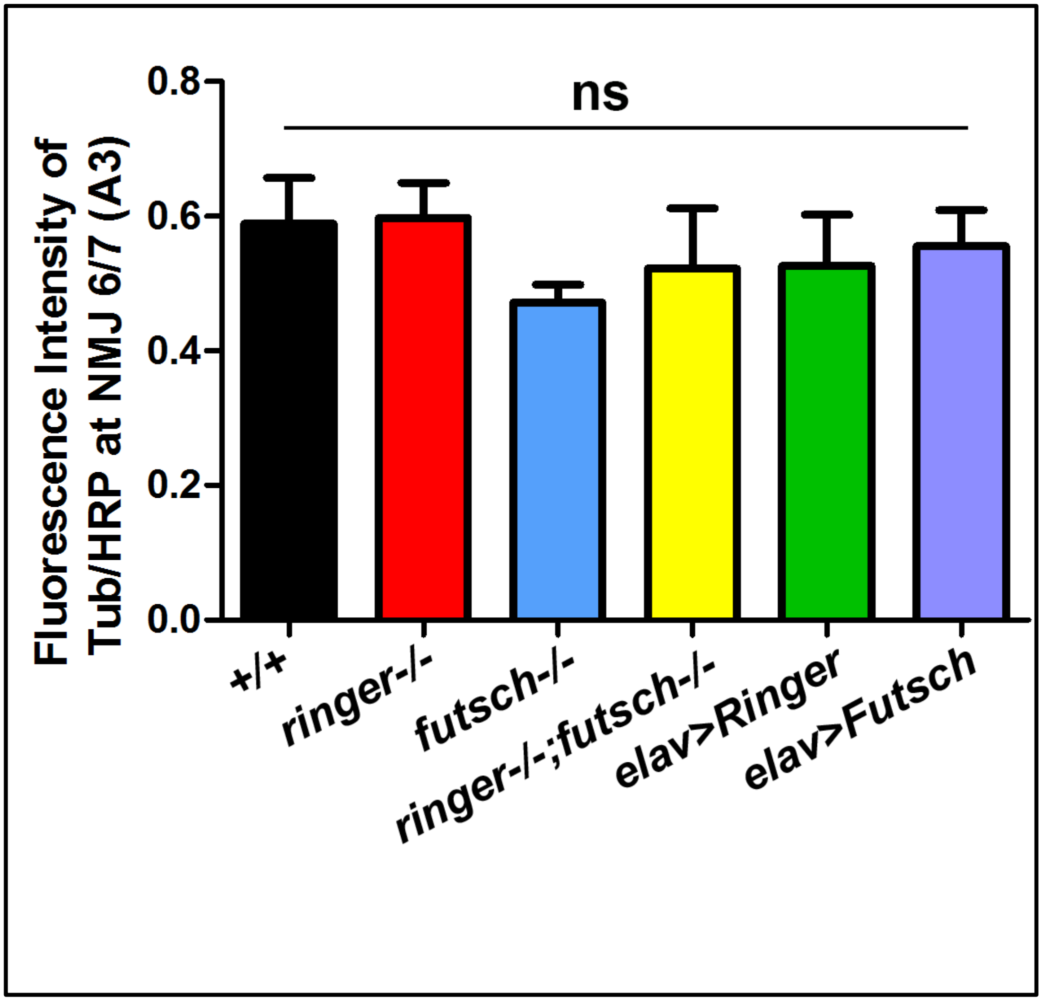

Supplementary Figure S3

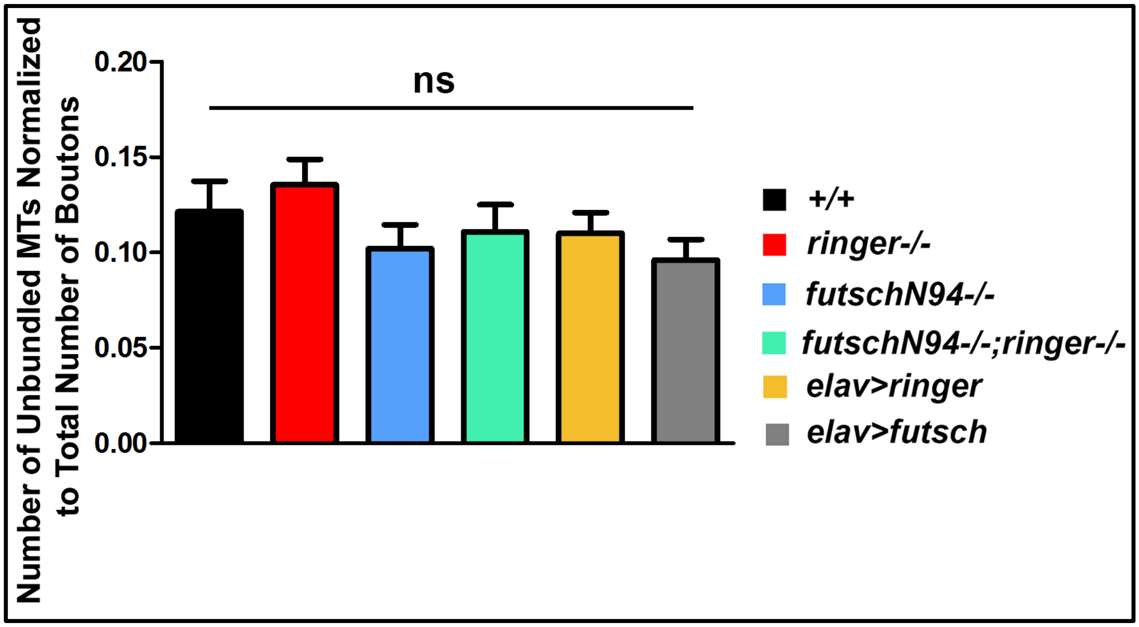

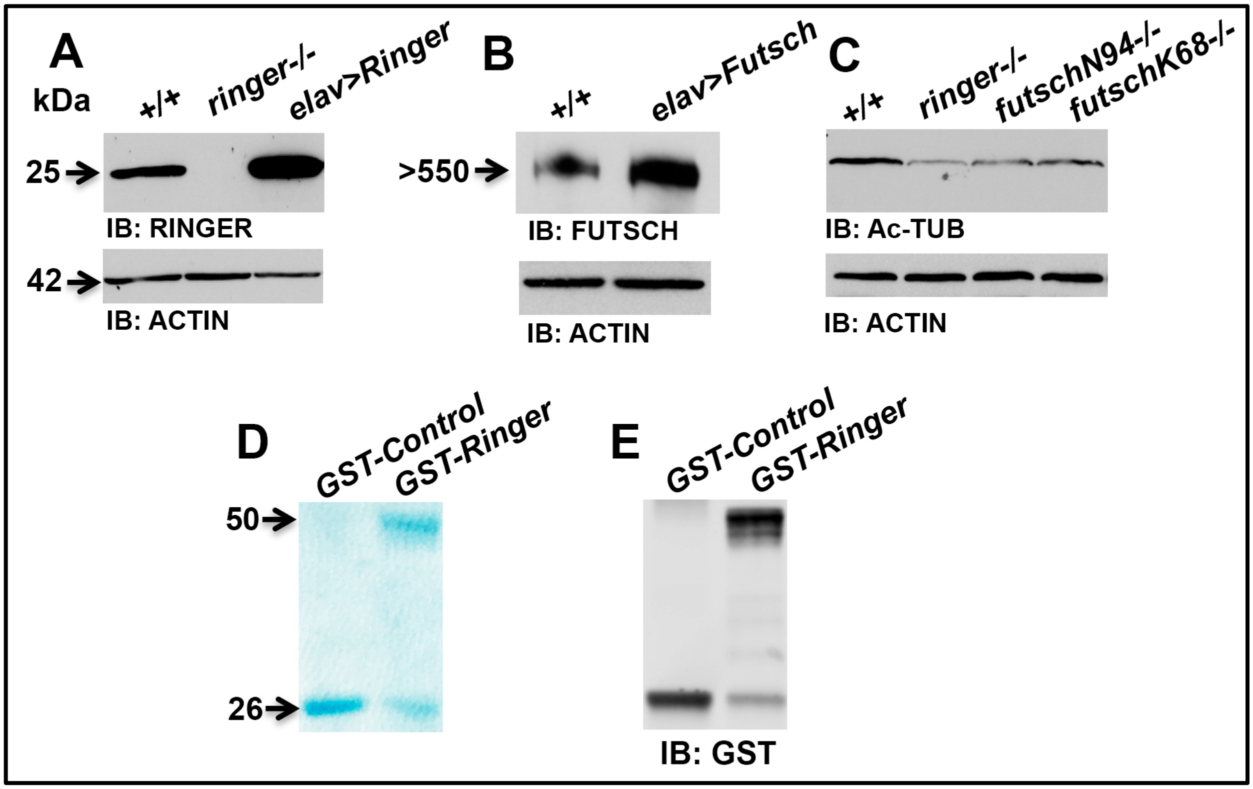
A
kDa
+/+
ringer-/-
elav>Ringer
25
IB: RINGER
42
IB: ACTIN
B
+/+
elav>Futsch
>550
IB: FUTSCH
IB: ACTIN
C
+/+
ringer-/-
futschN94-/-
futschK68-/-
IB: Ac-TUB
IB: ACTIN
D
GST-Control
GST-Ringer
50
26
E
GST-Control
GST-Ringer
IB: GST

Supplemental Table S1: Interacting Partners of TPPPs

Futsch/MAP1B	This study
Tubulin	This study; Hlavanda et al. 2002
GAPDH	Olah et al. 2006
LIMK1 and LIMK2	Acevedo et al. 2007; Heng et al. 2011
ERK2	Hlavanda et al. 2007
HDAC6	Tokeshi et al. 2010
α-Synuclein	Szunyogh et al. 2015
SIRT2*	Szabo et al. 2017

* Potential TPPP-interacting protein

Supplemental Legends

Supplementary Figure S1. Co-localization analyses of Ringer and Futsch.
(A, B) Coloc2 analysis was performed by specifying regions of interest (ROI) from confocal images of NMJ branches of wild type animals (a sample image shown in A) for studying colocalization of Ringer (red, A' A") with Futsch (green, A, A"). The spread of the data distribution showed an average of 70% colocalization using Pearson's correlation and 80% using Manders correlation (B).
Statistical significance was determined by one way ANOVA followed by post hoc Tukey's multiple comparison test and Student's t-test. Error bars represent mean $\pm$ SEM (***$p<0.001$, **$p\leq0.01$, *$p\leq0.05$, ns – not significant).
(Related to Figure 4).

Supplementary Figure S2. Synaptic Tubulin Levels Remain Unchanged in Single and Double Mutants of *ringer* and *futsch* and their Pre-Synaptic Overexpression.
Quantification of fluorescence intensity ratio of Tub/Hrp in represented genotypes.
Statistical significance was determined by one way ANOVA followed by post hoc Tukey's multiple comparison test and Student's t-test. Error bars represent mean $\pm$ SEM (***$p<0.001$, **$p\leq0.01$, *$p\leq0.05$, ns – not significant).
(Related to Figure 5).

Supplementary Figure S3. Unbundled Microtubules Remain Unchanged in Single and Double Mutants of *ringer* and *futsch* and their Pre-Synaptic Overexpression.
Number of unbundled microtubule loops normalized to total number of boutons in represented genotypes.
Statistical significance was determined by one way ANOVA followed by post hoc Tukey's multiple comparison test and Student's t-test. Error bars represent mean $\pm$ SEM (***$p<0.001$, **$p\leq0.01$, *$p\leq0.05$, ns – not significant).
(Related to Figure 6).

Supplementary Figure S4. Levels of Ringer, Futsch and Ac-Tub in Larvae.
(A-C) Immunoblots showing levels of Ringer (A), Futsch (B) and Ac-Tub (C) and their respective Actin controls from larval lysates of specified genotypes.
(D, E) Pull-down experiments from adult head lysates showing GST-Control (~26 kDa) and GST-Ringer (~50 kDa) stained with Coomassie (D) and probed with anti-GST antibodies (E).
(Related to Figure 7).

Supplemental References

Acevedo, K., Li, R., Soo, P., Suryadinata, R., Sarcevic, B., Valova, V.A., Graham, M.E., Robinson, P.J. and Bernard O. (2007). The phosphorylation of p25/TPPP by LIM kinase 1 inhibits its ability to assemble microtubules. *Exp Cell Res.* **313**, 4091-4106.

Heng, Y.W., Lim, H.H., Mina, T., Utomo, P., Zhong, S., Lim, C.T. and Koh C.G. (2012). TPPP acts downstream of RhoA-ROCK-LIMK2 to regulate astral microtubule organization and spindle orientation. *J Cell Sci.* **125**, 1579-90.

Hlavanda, E., Klement, E., Kókai, E., Kovács, J., Vincze, O., Tökési, N., Orosz, F., Medzihradszky, K.F., Dombrádi, V. and Ovádi, J. (2007). Phosphorylation blocks the activity of tubulin polymerization-promoting protein (TPPP): identification of sites targeted by different kinases. *J Biol Chem.* **282**, 29531-29539.

Oláh, J., Tokési, N., Vincze, O., Horváth, I., Lehotzky, A., Erdei, A., Szájli, E., Medzihradszky, K.F., Orosz, F., Kovács, G.G. and Ovádi, J. (2006). Interaction of TPPP/p25 protein with glyceraldehyde-3-phosphate dehydrogenase and their co-localization in Lewy bodies. *FEBS Lett.* **580**, 5807-5814.

Szunyogh, S., Oláh, J., Szénási, T., Szabó, A. and Ovádi, J. (2015). Targeting the interface of the pathological complex of α-synuclein and TPPP/p25. *Biochem Biophys Acta.* **1852**, 2653-2661.
